# A Perspective of International Collaboration Through Web-Based Telecommunication–Inspired by COVID-19 Crisis

**DOI:** 10.3389/fnhum.2020.577465

**Published:** 2020-11-23

**Authors:** Hamed Zaer, Wei Fan, Dariusz Orlowski, Andreas N. Glud, Anne S. M. Andersen, M. Bret Schneider, John R. Adler, Albrecht Stroh, Jens C. H. Sørensen

**Affiliations:** ^1^Department of Neurosurgery–Center for Experimental Neuroscience (CENSE), Aarhus University Hospital, Aarhus, Denmark; ^2^Leibniz Institute for Resilience Research, Mainz, Germany; ^3^Stanford University School of Medicine, Stanford, CA, United States; ^4^Zap Surgical Systems, Inc., San Carlos, CA, United States; ^5^Institute for Pathophysiology, University Medical Center, Mainz, Germany

**Keywords:** telecommunication, brain-computer interface (BCI), COVID-19, international collaboration, electrophysiology, brain electric activity, animal model, minipig model

## Abstract

The tsunami effect of the COVID-19 pandemic is affecting many aspects of scientific activities. Multidisciplinary experimental studies with international collaborators are hindered by the closing of the national borders, logistic issues due to lockdown, quarantine restrictions, and social distancing requirements. The full impact of this crisis on science is not clear yet, but the above-mentioned issues have most certainly restrained academic research activities. Sharing innovative solutions between researchers is in high demand in this situation. The aim of this paper is to share our successful practice of using web-based communication and remote control software for real-time long-distance control of brain stimulation. This solution may guide and encourage researchers to cope with restrictions and has the potential to help expanding international collaborations by lowering travel time and costs.

## Introduction

According to WHO, over 35 million people in 200 countries are affected by the COVID-19 pandemic (World Health Organization, 2020). The drastic increase of new cases led governments worldwide to introduce measures aimed at reducing health risks and controlling the dissemination of the disease. Because there is no objective criteria, by which to define acceptable risk for a given level of activity during the reopening, most countries chose to continue the lockdown in non-critical sectors. Social distancing, protective equipment, body temperature security checks, sanitation, disinfection of common and high-traffic areas, travel restrictions are included in the guidelines, which also have encouraged staff to do telework if possible to avoid infection (Prevention CfDCa, 2020).

Universities and their scientific work have generally been subject to these restrictions, resulting in the waste of precious funding resources and preparative work-hours on uncompleted experimental protocols. Sharing of innovative solutions with other researchers may help to overcome this pandemic-associated stoppage of biomedical research.

According to the theory of remote scientific collaboration (TORSC) advancing the future of scientific collaborations relies on new ideas in the science and capitalizing on tailored software (Olson et al., 2008). Achievements in efficient remote collaboration will positively influence science by inspiring and developing new collaboratories and new collaborative tools as well as learning and education. The word “collaboratory” was defined by William Wulf as “*a center without walls, in which the nation's researchers can perform their research without regard to a physical location, interacting with colleagues, accessing instrumentation, sharing data and computational resources, [and] accessing information in digital libraries*” (Lederberg and Uncapher, 1989). However, with the recent technological advancement, the trending paradigm is shifting from a just contextual or remote database collaboration toward a more practical online partnership such as telepresence. The researchers are capable of having a virtual physical existence in other labs. Recent advances in remote surgeries performed by robots under the remote control of surgeons who are not physically present represent the realization of this very concept (Tajima et al., 1999).

In this paper, we are presenting an international collaboration using tools including real-time long-distance control of brain stimulation, where groups of researchers from two different countries successfully collaborated online to complete the planned complex series of experimental surgeries and electrophysiology studies on miniature pigs (“minipigs”). This experimental concept overcomes several translational roadblocks, particularly the use of minipigs with brain sizes similar to humans, in stark contrast to the currently dominating studies in rodent models; this is important when using clinical radiosurgery tools in which the smallest beams would unacceptably envelop the brain of a rat. Our aim is to develop a clinically useful neuromodulation method that is non-invasive, focal, and durable. The International Neuromodulation Society defines neuromodulation as “the alteration of activity levels in neural tissue through targeted delivery of a stimulus, usually electrical.” In this way, activity in the targeted spot is “modulated” (Leksell and Backlund, 1978; Noren, 2004; Regis et al., 2010; Schneider and Adler, 2010). Neuromodulation may be applied directly to the actual location of a neurological problem (e.g., a seizure focus in epilepsy), to a node, or a location within a brain circuit wherein modulation of the target produces an intended network output from the circuit as a whole (e.g., subthalamic nucleus in Parkinson's disease). Radiosurgery has been proposed as a method of producing neuromodulation by Regis, who noted radiosurgery effects on epileptic tissue that otherwise seemed to retain basic neurological function (Regis et al., 2002, 2010). As proposed by Adler, the radiation-induced neuromodulation principle could be applied to targeted locations within known brain circuits for the treatment of refractory psychiatric and behavioral conditions (Schneider and Adler, 2010). When applied to a node or tract in a known brain circuit for intended effect, this is referred to as “radiomodulation.”

Radiomodulation remains a research hypothesis (Yeh et al., 2020). However, we need to understand in detail, whether these changes are long-lasting, and which components of the neuronal network are altered. The setup put forward in this remote collaboration aims for evaluating the radiomodulation effects on various neurophysiological parameters such as local gray matter excitability and the neural representation of sensory afferents.

The remote collaboration methodological concepts and tools that we discuss allowed us to carry out our experiment with the minimal number of staff during the surgeries, overcoming logistic issues such including closure of national borders.

### Technical Report

**Figure 1:**
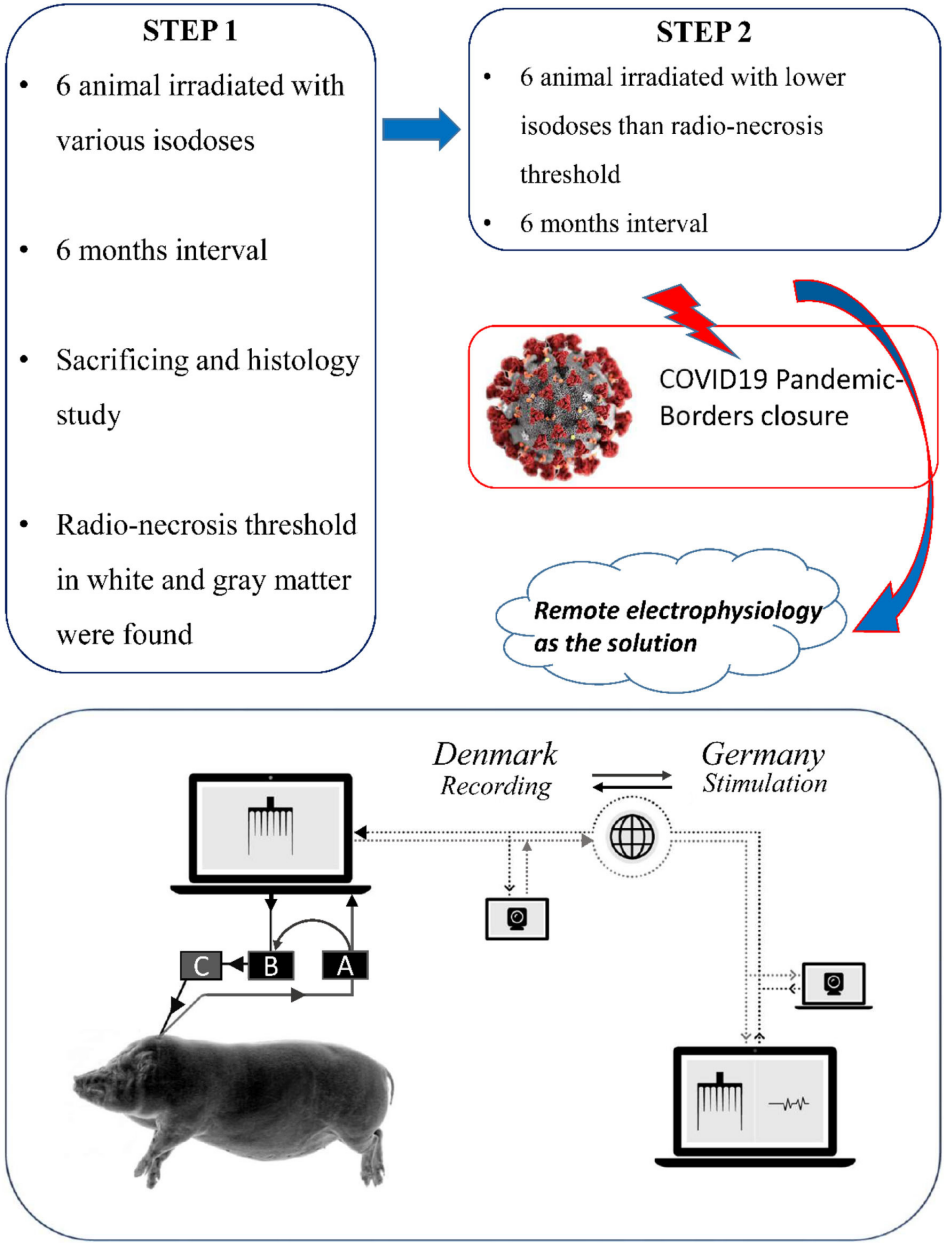
The full study design and the schematic figure of data flow: **(A)** PowerBox (recording) **(B)** CED-Box **(C)** Amplifier (stimulation).

**Figure 2:**
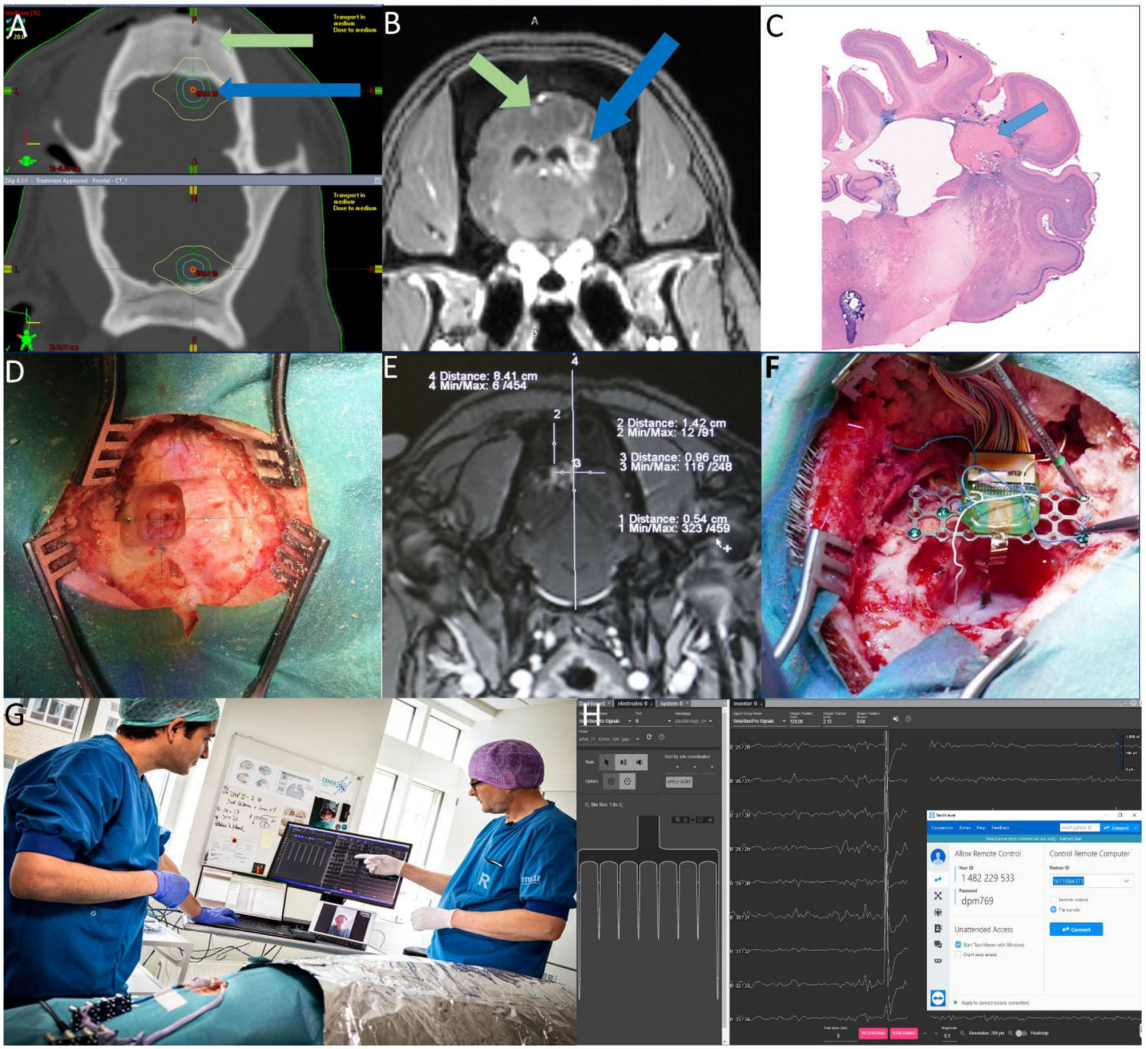
**(A)** Targeting MRI-CT fusion- Green arrow: Plastic fiducial screw- Blue arrow: Target area **(B)** Radio-necrotic lesions Green arrow: left cortex—Blue arrow: right white matter **(C)** Histology of Necrotic lesion in white matter **(D)** Alignment of titanium screws on the target area. **(E)** Calculation of the target on the contrast MRI. **(F)** Inserting and fixating the probe and Omnetic connector **(G)** The Jumpers are connected to the head-stage connected to the PC and electrophysiology devices and the animal is under the faraday cage. The electrophysiologist is communicating with the surgeons and controlling the electrophysiology software remotely (Photo by Tonny Foghmar). **(H)** Screenshot showing a schematic of the BCI chip implanted in the visual cortex and the stream of the electrophysiological activity in the Allego software with TeamViewer on top.

The project consisted of two phases (Figure 1). In the first phase, a group of six Göttingen minipigs underwent high-precision stereotactic radiosurgery to the cortex and immediately underlying white matter in the primary visual cortex (Zaer et al., 2020). Calculation of the target coordinates was carried out by implanting a copper-sulfate-filled fiducial marker in the skull followed by fused Magnetic Resonance Imaging (MRI) and Computerized Tomography (CT) scanning under general anesthesia (Figure 2A) (Glud et al., 2017; Lillethorup et al., 2018). After 6 months, the animals underwent 3D T1-weighted MRI brain scans (3.0 T Siemens Skyra−1 × 1 × 1 mm voxels, TE 3.7 ms, TR 2200 ms, TI 960 ms, FA 9 deg., 256 × 256 mm image size). The animals were then sacrificed to allow studies of the neuromodulatory effects of the stereotactic radiosurgery on small volumes of the brain tissue (Figure 2B). By performing histology, the radio-necrosis effect on the white and gray matter was distinguished (Figure 2C). The project's second phase entailed seven animals, which were irradiated before the COVID-19 crisis. The same method was used, but with lower isodoses in the right visual cortex (V1) to investigate the radio-biological responses of the neural tissue at doses below the threshold of tissue destruction. This was studied by implantation of an invasive intracortical multichannel electrode probe and a brain-computer interface into the target area to perform an electrophysiological readout. Due to lower isodoses, the lesion was not likely to be visible in the follow-up MRI, and for this reason, three titanium screws were put into the skull anterior, posterior, and lateral to the plastic fiducial marker as landmarks for more accuracy to find the target of the implantation surgery after 6 months post-irradiation (Figure 2D). Endpoint data, in the form of electrophysiological recordings from the probe, were to be obtained 6 months post-irradiation to allow latent radiation-associated biological changes in the tissue to occur. When the borders of Denmark were closed during the early months of 2020, a logistical challenge was immediately apparent: the neurophysiologists based in Germany could not be present alongside the neurosurgical team to secure the data acquisition. The experiment was thus at risk of being indefinitely postponed.

To remedy the situation, the Danish neurosurgery team connected the probe and recording devices in the manner specified by the instruction manuals, while real-time data receipt and stimulation triggering was installed in Germany (Figure 1 Schematic view). The German electrophysiologists then checked the whole setup online by Skype® video communication and a dry run was carried out the day before the first surgery.

In this report, we will not focus on the result of the main scientific project but the methodological concept of this online collaboration inspired by the logistic challenges due to the COVID-19 situation. On all surgery days, the surgical team consisted of three persons: a surgeon, an assistant surgeon, and an anesthesia technician. Animals were pre-medicated intramuscularly with 0.8 mg/kg midazolam and S-ketamine (20 mg/kg). Ear veins were catheterized (21G venflon) and anesthesia was induced with a second IV injection of Midazolam/S-ketamine. Shortly afterward, the minipigs were intubated (6.0 mm-tube) and anesthesia was maintained using 2% isoflurane through mechanical ventilation (Alstrup and Smith, 2013). The scalp was opened and a plastic fiducial marker was screwed into the cranium left to the midline. The animals then underwent MRI without and with 0.3 milliliters per kg Gadovist® (Gadolinium). The radio-necrosis in higher doses could clearly be seen in the MRI scanning (Figure 2E). The coordinates of the target area (radio-necrosis) in relation to the fiducial marker were calculated (Figure 2E). The scalp was reopened to the sides. By drilling off the superficial skull bone, the three pre-implanted titanium screws could be seen (Figure 2D). Coordinates of the target according to the plastic fiducial marker were completely in-line with the cross between the pre implanted titanium screws (Figure 2D). Confirming the alignment of the radio-necrosis site according to the pre-implanted screws in higher doses animals with the visible lesion after 6 months was of great importance since the MRI was not likely to visualize the lesion effect in lower isodoses. After opening the skull and dura on the V1 visual cortex, the probe consisting of 64 channels (16 for stimulation and 48 for recording, custom, Neuronexus, Inc. Ann Arbor, Michigan, USA) was inserted (Figure 2F). The probe was then fixed with fibrin sealant (TISSEEL® ^*^Baxter B. V., Utrecht, Netherlands) in the burr hole (Figure 2F). The epoxied Omnetics connector (Omnetics Connector Corporation, Minneapolis, Minnesota, USA) to the titanium plate was attached to the skull with titanium screws (Figure 2F). Electrophysiology reference wires were inserted in the white matter, under the titanium screws of the titanium plate, soft connective tissue of the scalp, and finally in the muscles. After semi-closing the surgical incision, the outgoing jumpers from the probe was connected to the head stage and Powerbox® (Neuronexus, Inc. Ann Arbor, Michigan, USA Figure 2G) to record the electrophysiological activity of the brain. The stimulation isolation unit ISO-STIM-01D and CED box MICRO3-1401 were connected to the PC for stimulation. The animal was then connected to the ground box as well as electrophysiology and anesthesia devices in the operation room and was covered with an aluminum box functioning as a faraday cage. We used the university hospital WiFi network with the security type of WPA2-Enterprise with 5 GHz bandwidth, 300 Mbps download, and an average upload rate of 200 Mbps, and a ping of 7 ms for the connection between Denmark and Germany. Then electrophysiologists from Germany took over the control of the connected PC online at the operation room by using remote control software (TeamViewer®). Remote electrophysiology recording was done by Allego® software and stimulation by Spike2® software. Both recordings on 48 channels and stimulation of the cortex and white matter on 16 channels were done online and remotely from Germany. We were able to record comprehensive electrophysiological alteration of the brain under light stable anesthesia in terms of gray matter excitability (gray matter stimulation), and the representation of visual afferents, the Visual Evoked Potential (VEP) (Figure 2H. Screenshot showing a schematic of the BCI chip implanted in the visual cortex and the stream of the electrophysiological activity). During the recording sessions, the two groups were communicating online throughout the entire procedure as if they were in the same room without any delay in the scheduled protocol and program (Figure 2G). A few simple verbal cues from the electrophysiologist to the surgical group regarding changing the stimulation headstage socket or turning on/off the amplifier were sufficient to run the experiment smoothly. Otherwise, all adjustments, stimulation, and recordings were done remotely from Germany. With the mentioned network properties, we did not face any software or connection disruptions during the sessions. Vital status, perfusion, and oxygenation were controlled by the ventilator and digital pulse-oximeter during the around 5 h of electrophysiological stimulation and recording per animal without any instability in these physiological parameters. All the animals went through the same procedures and were euthanized after the recording sessions following by the removal of the brains for histology analysis. The terabytes of electrophysiology data were saved on the university file transfer protocol (FTP) server and subsequently transferred to Germany for analysis by using the file transfer option of TeamViewer. However, even though we used a 300 Mbps fast fiber-optic network connection for uploading the data, we were mandated to leave the PCs on overnights to send the files benefiting from less network traffic facilitating data transfer. We did not face any kind of disturbance in data acquisition or transferring in terms of missing data or data corruption.

## Discussion

Scientific collaborations and international mobility have been directly linked to high-quality science and innovation with a great impact on scientific discovery, career development, and cultural maturity (Noren, 2004). Over the last 25 years, researchers have tried to build different computer-supported scientific collaboration environments, however, sustaining long-distance participation, solving larger-scale problems, and initiating breakthrough science have been achieved in a few numbers of these efforts (Bos et al., 2007). Competing priorities for members, limited funding, lack of long-term support, and *geographic dispersion* are still the main barriers to coordination and collaboration across research network members (Leroy et al., 2017). Improvements in communication technology, sharing expensive research equipment, increase in international citations showing the easier flow of knowledge across the world, and expanding the global research capabilities are mentioned as other benefits of the international collaborations (Wagner et al., 2015; Sohn, 2016). In contrast to the mentioned benefits, some recent studies anticipate that the innovation occurs mostly in centers, where innovators and scientists share office and lab space in clusters or have had close collaborations before (Sohn, 2016). The latter necessitates further efforts to facilitate the international collaboration by innovative solutions.

Most of the efforts so far have been focused toward facilitating of sharing the databases, distance education and tele-learning, computer-mediated communication, and cooperative/collaborative learning aspects of web-based collaborations and also behavioral experiments to gain access to large and diverse samples that would be difficult to get in a laboratory environment or institute (Sadler and Dooly, 2016; Anwyl-Irvine et al., 2020). Recently, new efforts in the clinic have focused on developing remote neuromodulatory interfaces for remote interaction between caregivers and patients. These include wireless deep brain stimulation (DBS) interfaces for Parkinson's disease and patient-controlled transcranial direct current stimulation (tDCS) devices for amyotrophic lateral sclerosis (ALS) patients. However, these devices are not designed for remote interaction between clinician and patient but is intended to aid the patient to adjust e.g., stimulus strength or pattern while at home, or in the case of tDCS, allow for at-home treatment. Indeed, the next step would entail the remote control of these devices by the clinician (Li et al., 2017; Sivaramakrishnan et al., 2019).

In the novel situation during the COVID-19 pandemic, a variety of social, business, and academic activities have been complicated or stopped by practical boundaries. Among them, scientific experiments are adversely influenced by lockdown, social distancing, and the required personal protection equipment and procedures. Maintaining the productivity of research groups and completing ongoing projects requires that we find creative solutions. One potential outcome is to be found in the generous sharing of data between collaborators. However, this pandemic forces us to become more practical in our collaboration in lieu of an evenhanded data transferring/sharing. In this manner, researchers from different institutes by applying carefully selected telepresence tools that enable remote control of research equipment as well as communication may make the difference between success and failure. The collaboration may fully benefit from its members and their respective experiences and expertise, and save on travel expenses. In the future, we look forward to advances in physical telepresence experiences in which communication and remote control are yet more fluidly integrated (Leithinger et al., 2014).

In this study, we managed to secure data from a complex large-animal study on time with the minimal number of staff physically present. The consistency of the results was retained and an international collaboration kept unperturbed and even the travel time and expenses saved. Coping with mass data storage and transferring issues, improvement in using the electronic infrastructure in scientific experiments, lowering travel expenses as the known facilitators of international collaborations (Barjak et al., 2013) are addressed in this short report and may help to expand the global scientific networks in the future (Leydesdorff et al., 2013).

The main contribution of our approach to the community represents the synergistic employment of both telecommunication and electrophysiological control software, yielding an active interaction of not-physically present scientists “tele-presence,” and an online real-time stimulation and recording of neuronal networks across the two countries.

This methodological concept can also be applied once the present COVID pandemic has passed, enabling more efficient international collaborations with lower costs. This is of particular importance for improving the participation and integration of low-income countries in the scientific community.

In conclusion, the COVID-19 outbreak has influenced all aspects of our life, including scientific experiments. Innovative solutions should be shared between researchers to cope with the negative consequences. Focusing on the remote control of experimental devices, and web-based communication can be one of the major solutions to maintain the experiments with the lowest physical contacts avoiding the unnecessary risk of infection distribution with the potential of consolidating the international collaborations even after the pandemic.

## Data Availability Statement

The raw data supporting the conclusions of this article will be made available by the authors, without undue reservation.

## Ethics Statement

The animal study was reviewed and approved by The Danish Animal Experiments Inspectorate in compliance with the ARRIVE guidelines and the 2010/63/EU directive for animal experiments. Written informed consent was obtained from the individual(s) for the publication of any potentially identifiable images or data included in this article.

## Author Contributions

HZ: writing the manuscript and performing the experiment (Electrophysiology and Surgery). WF: performing the experiment (Electrophysiology). DO: performing the histology. AG: assisting on the surgical procedure. AA: writing and editing the manuscript. MS and JA: design and conceptual assistance and editing the manuscript. AS: design and performing the experiment (Electrophysiology). JS: design, managing, and performing the experiment (surgical part). All authors approved the final version to be published.

## Conflict of Interest

MS and JA are employed by Zap Surgical Systems Inc., which financed the study, own stock, and have patents in the field. ZAP surgical system besides financing the experiment has not influenced the conclusions of the results presented in this paper. The remaining authors declare that the research was conducted in the absence of any commercial or financial relationships that could be construed as a potential conflict of interest.
